# 

**Published:** 1869-04

**Authors:** 


### CONTENTS:

### ORIGINAL COMMUNICATIONS.

ARTICLE I.—Valedictory Address to the Graduating Class of the Baltimore College of Dental Surgery. By Prof. E. Lloyd Howard, M.D. ... 554

ARTICLE II.—Studeuts Address. By George Fisk Keesee, D.D.S. ... 565

ARTICLE III.—Notes from Dental Practice. Filling Teeth ... 571

Alveolar Abscess with Facial Fistulæ ... 573

Methods for Determining the Size of the Roots of Teeth. By Mr. O. Salomon ... 574

ARTICLE IV.—Dental Education. By Richard B. Winder, D.D.S. ... 575

ARTICLE V.—Commencement Week at the Baltimore College of Dental Surgery ... 581

### CORRESPONDENCE.

ARTICLE VI.—Letter from Wm. O Kulp, D.D.S. ... 585

ARTICLE VII.—Pennsylvania College of Dental Surgery ... 586

ARTICLE VIII.—Philadelphia Dental College ... 587

ARTICLE IX.—Washington University of Medicine ... 587

### SELECTED ARTICLES.

ARTICLE X.—Hypodermic Medication. By Prof. Edward Warren, M.D. ... 588

ARTICLE XI.—The "Adulteration of Liquors." ... 590

### MONTHLY SUMMARY.

Employment of Glycerine Tannin ... 594

Mode of Dividing Glass ... 595

Steel ... 596

Fracture by Ointment ... 596

Early Dentition ... 597

Extraordinary Cure of Epilepsy ... 597

The Stains of Iodine ... 598

Second Sight ... 598

### BIBLIOGRAPHICAL NOTICE.

Injuries and Diseases of the Jaws. By Christopher Heath, F.R.C.S. ... 598

### EDITORIAL DEPARTMENT.

The Close of the II. Volume ... 599

The Annual Circular of the Baltimore College of Dental Surgery ... 599

Dr. L. Stuck's Rnbber Plates ... 600

Dr. McClelland's Rose-Pearl Base ... 600

